# Empirical analysis shows reduced cost data collection may be an efficient method in economic clinical trials

**DOI:** 10.1186/1472-6963-12-318

**Published:** 2012-09-15

**Authors:** Hildegard Seidl, Christa Meisinger, Rupert Wende, Rolf Holle

**Affiliations:** 1 Helmholtz Zentrum München – German Research Center for Environmental Health, Institute of Health Economics and Health Care Management, Munich, Germany; 2 Helmholtz Zentrum München – German Research Center for Environmental Health, Institute of Epidemiology, Munich, Germany; 3 Department of Internal Medicine I – Cardiology, Augsburg Hospital, Stenglinstraße 2, Augsburg, 86150, Germany; 4 MONICA/KORA Myocardial Infarction Registry, Augsburg Hospital, Stenglinstraße 2, Augsburg, 86150, Germany

**Keywords:** Method of data collection, Reduced cost data collection, Interpolation, Extrapolation, Sample size calculation, Elasticity of variance

## Abstract

**Background:**

Data collection for economic evaluation alongside clinical trials is burdensome and cost-intensive. Limiting both the frequency of data collection and recall periods can solve the problem. As a consequence, gaps in survey periods arise and must be filled appropriately. The aims of our study are to assess the validity of incomplete cost data collection and define suitable resource categories.

**Methods:**

In the randomised KORINNA study, cost data from 234 elderly patients were collected quarterly over a 1-year period. Different strategies for incomplete data collection were compared with complete data collection. The sample size calculation was modified in response to elasticity of variance.

**Results:**

Resource categories suitable for incomplete data collection were physiotherapy, ambulatory clinic in hospital, medication, consultations, outpatient nursing service and paid household help.

Cost estimation from complete and incomplete data collection showed no difference when omitting information from one quarter. When omitting information from two quarters, costs were underestimated by 3.9% to 4.6%.

With respect to the observed increased standard deviation, a larger sample size would be required, increased by 3%. Nevertheless, more time was saved than extra time would be required for additional patients.

**Conclusion:**

Cost data can be collected efficiently by reducing the frequency of data collection. This can be achieved by incomplete data collection for shortened periods or complete data collection by extending recall windows. In our analysis, cost estimates per year for ambulatory healthcare and non-healthcare services in terms of three data collections was as valid and accurate as a four complete data collections. In contrast, data on hospitalisation, rehabilitation stays and care insurance benefits should be collected for the entire target period, using extended recall windows. When applying the method of incomplete data collection, sample size calculation has to be modified because of the increased standard deviation. This approach is suitable to enable economic evaluation with lower costs to both study participants and investigators.

**Trial registration:**

The trial registration number is ISRCTN02893746

## Background

Economic evaluation alongside clinical trials is gaining importance because of demographic trends towards an ageing population, which is a relevant driver for resource use in healthcare [1-3]. There are different types of data that can be used to collect information on healthcare costs: reviewing medical charts or administrative data sets; drawing on experts’ experiences of healthcare utilisation; and gathering patients’ self-reported resource use [4]. Patients’ self-reported resource use can be recorded prospectively with cost diaries or retrospectively by means of questionnaires. However, cost data collection in clinical trials can be burdensome and can increase both the costs of clinical trials [5] and the burden on study participants [6] and clinical investigators [7].

For economic evaluation from a societal perspective, only patients’ self-reported resource use offers an account of out-of-pocket payments (e.g. paid household help or over-the-counter medications). Cost data collection from health insurance companies is complex because patients are enrolled in different insurance funds, which is true in Germany and also in the USA. Hence, prospective or retrospective methods based on patient recall are widely used [8-10].

Retrospective assessment of costs via questionnaires raises the question of how often participants should be contacted and what is the appropriate recall time frame [11,12]. Recall ability declines with age, the frequency of resource use and the length of the recall time frame [9]. If patients are asked more frequently, recall bias is minimised, but the cost of data collection and the burden on study participants are increased [13]. For the prospective assessment of costs via cost diaries, the period covered by each diary and the frequency with which the diaries are sent back needs to be defined. For long-term clinical trials, e.g. 12 months or longer, the burden on study participants of keeping the diary is substantial and it is unlikely that participants will complete all the diaries [14].

With regard to the above-mentioned problems, an alternative approach to minimise recall bias and the frequency of questioning is to limit the time period covered by each diary or to limit the recall period [15]. As a consequence, gaps in survey periods arise and must be filled appropriately by the collected data. This approach has already been used to collect cost data in both cross-sectional studies and randomised controlled trials. In a cross-sectional study on healthcare costs in the elderly, Heinrich et al. assessed resource use over various time spans and extrapolated the data to annual costs by assuming that the documented resource use over abbreviated periods would also be found over a 12-month period [16]. In the randomised controlled trial on the cost-utility of psychological treatment for depression and anxiety, Hakkaart-van Roijen et al. [17] interviewed patients every 3 months over a period of 1.5 years asking for medical resource use over the past 4 weeks. They extrapolated the documented resource use by assuming that 4 weeks was representative of the total period of 3 months. Kimman et al. [18] conducted an economic evaluation alongside the randomised controlled trial on treatment for breast cancer, collecting cost data after 3, 6 and 12 months for a period of 4 weeks to interpolate them by assuming that 4 weeks was representative of the in-between periods.

Goossens et al. [14] investigated whether there is a difference in costs between data from limited time periods extrapolated to 1 year and cost diary data from the entire year. They could not find any significant difference between the cost data in the alternative periods. Clarke et al. [13] published a statistical framework specifying cases for which the recall period can be limited by trading off recall bias against information loss.

Considering the importance of limiting the recall period or the period covered by each diary in favour of reduced time and effort for both participants and investigators, there is still little empirical evidence regarding the validity of cost collection with gaps in survey periods in long-term clinical trials. For this purpose, we compared the impact of different gaps in survey periods on the validity of cost measurement in the relevant healthcare service categories using data from a randomised controlled trial.

The main objective of our paper is to assess the validity of incomplete cost data collection concerning the precision and accuracy of cost estimates. Two further objectives are to identify the healthcare service categories suitable or not suitable for inter-/extrapolating incompletely collected cost data and to demonstrate the consequences of collecting data for an abbreviated time period on sample size calculation with respect to changing the variance of the estimate.

## Methods

### Study design and study population

The data used were obtained from the randomised controlled KORINNA trial (Koronarinfarkt-Nachbehandlung im Alter), which is an ongoing monocentre study at the Central Hospital of Augsburg, Germany, to evaluate the cost-effectiveness of a case management intervention by trained nurses in elderly patients (≥ 65 years) with acute myocardial infarction. The control group received usual care. The primary goal of the KORINNA study is to assess whether case management can reduce readmission or out-of-hospital death. As a secondary objective, the incremental cost-utility ratio of this case management intervention will be estimated. The intervention and observation period covers 1 year. The study protocol, which was approved by the Ethics Committee of the Bavarian Chamber of Physicians, describes the intervention in more detail and is published elsewhere [19]. The trial registration number is ISRCTN02893746. Between September 2008 and May 2010, 340 patients from the Augsburg region were enrolled. For this analysis, cost data for 234 patients with complete 1 year follow-up (quarters one, two, three and four) were available. The mean age of participants was 75 years, and nearly 38% (n=88) were women.

### Cost measurement

Cost measurement by bottom-up gross costing [20] is a two-step process. First, duration and/or frequency of resources used by patients were collected via a questionnaire-based interview. Second, unit prices are determined and multiplied by duration and/or frequency of resource use.

Cost measurement was performed from the societal perspective, which includes both direct healthcare costs and direct non-healthcare costs. Indirect costs are not considered in this analysis, as all participants were already retired and did not incur production loss due to illness-related absence from work. Data collection was not limited to disease-related services and protocol-driven costs were factored out [19,21].

#### Collection of resource use

Over a 1-year period, participants were interviewed quarterly regarding the previous time period of 3 months, either by computer-assisted telephone interview (CATI) or in a face-to-face interview. In the case of CATI, plausibility checks were included and, in the case of face-to-face interviews, double data entry was applied. Data evaluation and necessary adjustments were performed as described below.

##### Direct healthcare use

For costs incurred for outpatient and inpatient care, the number of visits to a general practitioner, specialist care, ambulatory clinics in hospital and physiotherapy, days spent in hospitals, intensive care units and rehabilitation were documented. Medication administered at the time of the survey was recorded by IDOM software, a database-supported identification system that logs the name, units, pharmaceutical identification number, time period, package size and price per package [22]. In certain cases, medication data had to be adjusted to calculate the cost of medication properly and this is described as follows. For some medications – such as insulin – package size was converted from millilitres or prefilled pens/syringes to international units. Similar problems arose in the case of drops, inhalants and aerosols. In these cases, the numbers of drops or puffs per package had to be calculated based on information from the pharmaceutical company because the units per package disagree with the units of medication administration. If patients ingested antibacterials or subcutaneously administered anticoagulants, it was assumed that one package of the medication was bought. Medications not taken regularly were not included in the analysis.

##### Direct non-healthcare use

Costs incurred for formal care and home help were documented as days per week and hours per day of outpatient nursing service and paid household help. The level of care needs, as assessed by the medical services of the long-term care insurance fund, served as a proxy for the extent of informal care [23].

#### Unit prices for resource use

Unit price calculation for resource use was primarily based on the methods published by Krauth et al. [24]. All unit prices were reported for the year 2008. Table 1 gives an overview of prices assigned to the resource quantities.

**Table 1:** Unit prices (all unit prices are expressed in euros at 2008 values)

**Resource category**	**Unit price in euros (2008)**		**Units**
Direct healthcare			
Physicians			
General practitioner	20.65	per	contact
Internist	44.44	per	contact
Orthopaedist	27.92	per	contact
Neurologist	18.30	per	contact
Ophthalmologist	31.17	per	contact
Otolaryngologist	29.64	per	contact
Gynaecologist	32.03	per	contact
Dermatologist	18.62	per	contact
Urologist	34.41	per	contact
Other	27.43	per	contact
Physiotherapist	13.75	per	contact
Ambulatory clinic in hospital	40.31	per	contact
Inpatient care/hospital	511.07	per	day
Inpatient care/intensive care unit	1199.14	per	day
Inpatient rehabilitation	100.00	per	day
Outpatient rehabilitation	1535.00	per	stay
Drugs	various	quantity - according to medication	
Direct non-healthcare			
Outpatient nursing service	28.30	per	hour
Paid household help	15.70	per	hour
Informal care			
care level			
none	0	per	month
1	215.00	per	month
2	420.00	per	month
3	675.00	per	month

The costs of physicians, published as weighted average values of patient-physician contact for privately and statutorily insured patients, [24] were updated concerning the reimbursement from the Statutory Health Insurance (SHI) per case, drawing on data from the National Association of Statutory Health Insurance Physicians [25]. The weighted costs per contact with physiotherapists (€13.75) were calculated from data from the SHI contract for the supply of remedies [26] and the stipulation of allowance for civil servants [27]. Costs of ambulatory clinics in hospital were calculated by the average reimbursement from the Augsburg hospital (€40.31). The price per hospital day (€511.07) was based on Federal Statistical Office [28] and National Health Report data [29], using Krauth’s [24] calculation method. The latest published price of intensive care units from 2003 [30] was extrapolated to 2008 using the yearly inflation rate of hospital costs [29] (€1,199.14). Inpatient rehabilitation (€100 per day) and outpatient rehabilitation (€1535 per case) were based on data from the pension insurance companies [31]. The prices of medications were based on the medication database of the scientific institute of the statutory sickness funds in Germany (WIDO) [32]. The total drug costs were computed by calculating cost per dose and multiplying by the frequency and duration of administration. Costs per hour of outpatient nursing service (€28.30) and paid household help (€15.70) were valued based on the contract between the associations of nursing caregivers and the Local Health Care Fund [33]. Informal care was valued according to the benefits received per nursing care level, which is granted by the German Long Term Care Insurance after assessing the required level of nursing care [23]. The benefit is a transfer payment to patients, who do not engage outpatient nursing services but also need help in activities of daily living (ADL), e.g. eating, bathing, dressing, and/or in instrumental activities of daily living (IADL), e.g. housework, cooking, shopping. The benefit level is determined by nursing care level and, although it is a transfer payment, it is used here as a proxy value for informal care.

### Dealing with missing data

As only data from complete follow-up (quarters one, two, three and four) were used, there were only a few itemwise missing values (96.2% of the patients had complete cost data), except for medication. Hence, for missing values mean imputation was employed by generating means of the observed data for the corresponding variables. Some 16% of medications did not have a valid pharmaceutical identification number. As unit costs are only available for medication with a pharmaceutical identification number, missing data were substituted based on the documented medication name. If no dosage was specified, it was replaced by the patient’s medication data from other quarters.

### Impact of different time periods on measurement of resource use and cost

The objective of our analysis was to investigate the validity of incomplete cost data collection in long-term clinical trials, using data from the KORINNA study. We did not consider excluding the first and the last quarter of data collection. The first quarter is important because response rates commonly dwindle after the initial assessment as a result of withdrawal of informed consent or death of study participants and, in the last period, cost data can be collected efficiently together with the final assessments. Accordingly, either the second and/or third quarter were excluded for our study.

For each resource category, mean costs per quarter and per capita were calculated based on quarterly data collected from 1 year. The difference between quarterly costs over the course of time was compared by repeated measures analysis of variance and Tukey’s multiple comparison test. To determine the resource category suitable for incomplete data collection, mean differences between quarters were assessed, and assumed inherent recall bias was considered. In the case of minor recall bias, complete data collection by extending recall windows was preferred to incomplete data collection.

For resource categories suitable for incomplete data collection, different kinds of cost calculations were employed. For extrapolation, the complete values from the following quarter replaced those from the missing quarter. For interpolation, the means of the individual values from the previous quarter and the following quarter were used to replace the values from the missing quarter.

**Figure 1:**
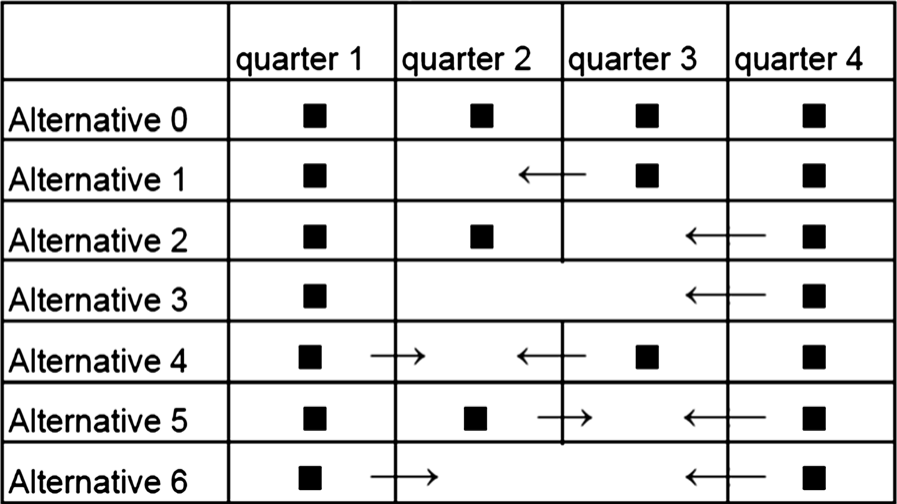
**Overview of incomplete cost collection: methods and omitted quarters. **Alternative 0: complete cost collection. Alternative 1: extrapolation, omitted quarter 2, replaced by quarter 3. Alternative 2: extrapolation, omitted quarter 3, replaced by quarter 4. Alternative 3: extrapolation, omitted quarters 2 and 3, replaced by quarter 4. Alternative 4: interpolation, omitted quarter 2, replaced by quarters 1 and 3. Alternative 5: interpolation, omitted quarter 3, replaced by quarters 2 and 4. Alternative 6: interpolation, omitted quarters 2 and 3, replaced by quarter 1 and 4.

For each method (extrapolation and interpolation), three alternative periods of data collection were considered (Figure 1):

• Alternative 1 (Alt 1): Data collection in the first, third and fourth quarters and replacing the data from the second quarter by extrapolating the data from the third quarter

• Alternative 2 (Alt 2): Data collection in the first, second and fourth quarters and replacing the data from the third quarter by extrapolating the data from the fourth quarter

• Alternative 3 (Alt 3): Data collection in the first and fourth quarters and replacing the data from the second and third quarters by extrapolating the data from the fourth quarter

• Alternative 4 (Alt 4): Data collection in the first, third and fourth quarters and replacing the data from the second quarter by interpolating the data from the first and third quarters

• Alternative 5 (Alt 5): Data collection in the first, second and fourth quarters and replacing the data from the third quarter by interpolating the data from the second and fourth quarters

• Alternative 6 (Alt 6): Data collection in the first and fourth quarters and replacing the data from the second and third quarters by interpolating the data from the first and fourth quarters

We analysed the influence of the chosen methods on the accuracy and precision of the total cost estimate in the following way.

Cost data were calculated as mean costs, and the differences between complete and incomplete cost data collection were tested using standard errors and p-values from paired t-tests. Alternatively, bootstrap resampling was applied to compute p-values, as there was the possibility that the differences in costs were skewed. We drew 1000 re-samples of the data set with replacement.

The effects of alternative intensity of data collection on precision were studied by comparing the standard deviation of the respective estimates.

Larger sample sizes are required because of an increase in the variance of the estimate arising from a shorter period of data collection to keep the power. This effort must be weighed against the advantages of reducing the data collection costs and the burden on study participants.

Based on the equations for sample size calculation for a single outcome and for cost-effectiveness, [34] the impact of a 1% increase in standard deviation on percentage change in sample size, called elasticity (ε), was calculated assuming ceteris paribus, where α, β and the expected mean difference in costs are not changed The details of the mathematical calculations are shown in Appendix 1. The amount of time that would be saved by collecting data only for a limited period is compared with the consumed time for additional patients as a result of the requirements of a larger sample size.

All analyses were performed with SAS (Version 9.2, SAS-Institute Inc., Cary, NC, USA).

## Results

### Resource use

Table 2 provides an overview of mean resource use per patient over 12 months.

**Table 2:** Mean resource use (in number of contacts unless stated otherwise) per patient over 12 months, n=234

**Resource category**	**Overall means**	**(SD)**	**Resource use (%)**	**Means of patients using services (SD)**	
Direct healthcare					
General practitioner	13.26	(22.46)	99.37	13.32	(11.45)
Internist	2.16	(2.87)	67.95	3.18	(2.89)
Orthopaedist	1.40	(3.03)	33.33	4.21	(3.99)
Neurologist	0.28	(0.85)	12.38	1.83	(1.36)
Ophthalmologist	1.46	(2.79)	47.15	3.16	(3.39)
Otolaryngologist	0.41	(1.18)	19.66	2.07	(1.94)
Gynaecologist	0.15	(0.58)	9.40	1.64	(1.09)
Dermatologist	1.09	(3.40)	28.63	3.81	(5.51)
Urologist	0.49	(1.38)	18.38	2.67	(2.16)
Other	0.35	(1.33)	14.10	2.49	(2.72)
Sum of physicians	21.06	(14.60)	99.37	21.15	(14.56)
Physiotherapist	5.03	(11.82)	32.05	15.71	(16.44)
Ambulatory clinic in hospital	0.89	(2.75)	32.48	2.74	(4.28)
Inpatient care in stays	0.85	(1.32)	44.87	1.90	(1.37)
Inpatient care/hospital in days (without intensive care unit)	7.89	(16.56)	44.87	17.59	(21.02)
Inpatient care/intensive care unit in days	0.51	(1.74)	14.10	3.64	(3.23)
Inpatient/outpatient rehabilitation in days	13.86	(13.06)	59.40	23.33	(8.09)
Drugs in numbers of medication taken at the same time	6.99	(2.15)	100.00	6.99	(2.15)
Direct non-healthcare					
Outpatient nursing service in hours	5.14	(20.98)	7.69	66.81	(40.90)
Paid household help in hours	26.65	(156.40)	10.68	249.48	(423.74)
Informal care (%)					
without care level			92.74		
Care level one			5.89		
Care level two			1.28		
Care level three			0		

Within 1 year, almost all subjects consulted a general practitioner and ca. 68% consulted a specialist in internal medicine. On average, patients consulted a physician 21 times a year. All patients were on medication and ingested on average seven different types of medication at the same time. Approximately one third of subjects received physiotherapy about 16 times over the year and attended the ambulatory clinic at hospital about three times a year. Forty-five per cent of patients were hospitalised and admitted for an average of 19 days; about one third of them had a stay in the intensive care unit of 3.6 days on average. The majority (59%) of patients received rehabilitation. About 8% used the outpatient nursing service for 1.3 h per week, and ca. 11% employed paid household help for 4.8 h per week. Approximately 93% of the patients were without a care level; about 6% were classed as care level one, 1% as care level two and none as care level three.

### Resource categories suitable for incomplete data collection

As presented in Table 3, the Tukey test showed that the costs of inpatient care, rehabilitation and care insurance benefit levels as a proxy for informal care were significantly different between quarters. Usage of physiotherapy, ambulatory clinic in hospital, outpatient nursing service and paid household help did not differ significantly between quarters (Table 3). Costs of physicians were significantly lower in the first quarter than in subsequent quarters. Costs of medication were significantly lower in the fourth quarter than in the second and third quarters.

**Table 3:** Mean costs per patient over different quarters, n=234

	**Period of time**								**Significant difference between quarters**
	**Quarter 1**		**Quarter 2**		**Quarter 3**		**Quarter 4**		
**Cost category**	**Means**	**(SD)**	**Means**	**(SD)**	**Means**	**(SD)**	**Means**	**(SD)**	
Direct healthcare costs									
Physicians	115.00	(94.54)	139.14	(145.99)	133.33	(119.93)	135.91	(108.93)	**1 vs. 2; *1 vs. 4
Physiotherapist	13.57	(49.18)	19.57	(57.59)	16.98	(48.73)	19.10	(57.80)	
Ambulatory clinic in hospital	5.17	(20.14)	7.92	(37.99)	14.99	(84.45)	7.75	(32.38)	
Inpatient care	1436.71	(4175.58)	1529.02	(4774.59)	723.35	(2851.91)	959.82	(3070.25)	*2 vs. 3
Inpatient/outpatient rehabilitation	1238.61	(1085.16)	68.38	(405.57)	24.51	(217.36)	52.56	(365.25)	**1 vs. 2, 1 vs. 3, 1 vs. 4
Drugs	325.58	(178.80)	339.94	(203.90)	337.11	(200.37)	312.89	(204.51)	**4 vs. 2, 4 vs. 3
Sum of direct healthcare costs	3134.64	(4271.98)	2104.00	(4898.88)	1250.28	(3010.82)	1488.07	(3270.83)	**1 vs. 2, 1 vs. 3, 1 vs. 4, 2 vs. 3
Direct non-healthcare costs									
Outpatient nursing service	34.67	(162.79)	43.50	(237.95)	38.70	(181.42)	28.56	(151.73)	
Paid household help	96.38	(643.07)	111.01	(684.75)	109.03	(626.12)	102.05	(645.84)	
Informal care	35.45	(177.98)	43.72	(190.73)	46.47	(194.71)	54.74	(205.98)	**1 vs. 4
Sum of direct non-healthcare costs	166.49	(710.79)	198.21	(811.39)	194.20	(729.73)	185.35	(743.39)	
Sum of costs	3301.14	(4353.64)	2302.21	(5068.35)	1444.50	(3197.00)	1673.42	(3402.68)	**1 vs.2, 1 vs. 3, 1 vs. 4, 2 vs. 3

*p-value of Repeated Measure ANOVA (RMANOVA) Tukey test <0.10.

**p-value of Repeated Measure ANOVA (RMANOVA) Tukey test <0.05.

### Validity of incomplete cost collection

For the cost categories physiotherapy, ambulatory clinic in hospital, outpatient nursing service, paid household help, visits to physicians and medications, complete cost collection was compared with incomplete cost collection to ascertain which period of data collection could be omitted.

Comparisons of complete cost collection per year and per capita vs. three alternative periods of incomplete data collection using extrapolation are illustrated in Table 4.

**Table 4:** Complete cost collection vs. incomplete cost collection and extrapolation (by the respective quarter), n=234

	**Complete cost collection**	**Incomplete cost collection**												
		**Extrapolation**												
		**Omitted quarters**												
	**Alt 0**^**a**^		**Alt 1**^**b**^				**Alt 2**^**c**^				**Alt 3**^**d**^			
			**Quarter 2 (by 3)**				**Quarter 3 (by 4)**				**Quarter 2 and 3 (by 4)**			
**Cost category**	**mean**	**(SD)**	**mean**	**(SD)**	**mean difference**	**(StdErr)**	**mean**	**(SD)**	**mean difference**	**(StdErr)**	**mean**	**(SD)**	**mean difference**	**(StdErr)**
Direct healthcare costs														
Physicians	523.38	(358.27)	517.57	(365.32)	5.81	(9.51)	525.99	(370.53)	2.62	(6.67)	522.75	(377.53)	0.63	(11.91)
Physiotherapist	69.29	(162.63)	66.69	(166.77)	2.61	(3.78)	71.38	(178.98)	2.09	(3.33)	70.91	(202.65)	1.62	(6.73)
Ambulatory clinic in hospital	35.79	(110.83)	42.89	(181.93)	7.10	(6.00)	28.60	(83.44)	7.19	(5.30)	28.41	(99.47)	7.38	(6.20)
Drugs	1315.52	(707.38)	1312.68	(721.69)	2.84	(7.97)	1291.31	(709.17)	**24.21	(7.54)	1264.26	(739.52)	**51.26	(16.33)
Sum of direct healthcare costs	1943.99	(936.67)	1939.82	(946.22)	4.18	(14.01)	1917.28	(962.29)	**26.71	(12.04)	1886.32	(997.06)	**57.67	(22.20)
Direct non-healthcare costs														
Outpatient nursing service	145.44	(593.85)	140.63	(569.41)	4.81	(10.17)	135.29	(573.69)	10.15	(9.10)	120.36	(549.63)	25.08	(19.36)
Paid household help	418.46	(2455.42)	416.48	(2433.88)	1.97	(20.61)	411.48	(2489.45)	6.97	(23.66)	402.53	(2527.39)	15.93	(45.70)
Sum of direct non-healthcare costs	563.89	(2568.91)	557.11	(2534.07)	6.78	(22.98)	546.77	(2589.39)	17.12	(24.27)	522.89	(2598.16)	41.00	(46.89)
Sum of costs	2507.88	(2864.38)	2496.93	(2827.43)	10.96	(28.11)	2464.05	(2882.21)	43.83	(27.12)	2409.21	(2893.05)	*98.67	(54.67)

*p-value of paired *t*-test/bootstrapping of differences between complete and incomplete cost collection <0.10.

**p-value of paired *t*-test/bootstrapping of differences between complete and incomplete cost collection <0.05.

^a^Alternative 0, complete cost collection.

^b^Alternative 1, incomplete cost collection, omitted quarter 2, replaced by quarter 3.

^c^Alternative 2, incomplete cost collection, omitted quarter 3, replaced by quarter 4.

^d^Alternative 3, incomplete cost collection, omitted quarters 2 and 3, replaced by quarter 4.

All comparisons between complete and incomplete data collection were not significantly different regarding the p-values of paired t-tests or the p-values of bootstrapping the differences in costs. Only drug costs, which averaged €1315.52 in complete data collection, differed significantly from incomplete data collection when applying Alt 3 (€1264.26) and Alt 2 (€1291.31). That was to be expected as the mean costs of the second and third quarters differed significantly from the mean costs of the fourth quarter (Table 3). By extrapolating the costs of the third quarter to the second quarter (Alt 1), cost estimation per year showed no significant differences in means.

Comparisons of complete and incomplete data collection using interpolation are shown in Table 5.

**Table 5:** Complete cost collection vs. incomplete cost collection and interpolation (by the respective quarters), n=234

	**Complete cost collection**	**Incomplete cost collection**												
		**Interpolation**												
		**Omitted quarters**												
	**Alt 0**^**a**^		**Alt 4**^**b**^				**Alt 5**^**c**^				**Alt 6**^**d**^			
			**Quarter 2 (by 1 and 3)**				**Quarter 3 (by 2 and 4)**				**Quarters 2 and 3 (by 1 and 4)**			
**Cost category**	**means**	**(SD)**	**means**	**(SD)**	**means in difference**	**(StdErr)**	**means**	**(SD)**	**means in difference**	**(StdErr)**	**means**	**(SD)**	**means in difference**	**(StdErr)**
Direct healthcare costs														
Physicians	523.38	(358.27)	508.40	(343.27)	14.97	(8.58)	527.56	(383.66)	4.19	(7.03)	501.87	(344.89)	21.51	(11.62)
Physiotherapist	69.29	(162.63)	64.97	(164.25)	4.31	(3.23)	71.61	(175.50)	2.32	(2.83)	65.37	(185.64)	3.92	(5.42)
Ambulatory clinic in hospital	35.79	(110.83)	37.93	(142.50)	2.14	(3.66)	28.62	(84.32)	7.18	(5.45)	25.88	(76.71)	*9.91	(5.88)
Drugs	1315.52	(707.38)	1306.92	(701.52)	8.60	(7.23)	1304.79	(707.09)	*10.72	(5.75)	1276.92	(692.75)	**38.60	(12.53)
Sum of direct healthcare costs	1943.99	(936.67)	1918.23	(917.58)	**25.77	(11.82)	1932.58	(962.72)	11.41	(10.89)	1870.04	(936.79)	**73.95	(19.96)
Direct non-healthcare costs														
Outpatient nursing service	145.44	(593.85)	138.62	(546.78)	6.82	(11.98)	142.76	(608.39)	2.67	(7.35)	126.47	(537.84)	18.97	(20.85)
Paid household help	418.46	(2455.42)	410.15	(2442.17)	8.30	(21.42)	415.96	(2494.90)	2.50	(17.83)	396.86	(2506.58)	21.60	(39.02)
Sum of direct non-healthcare costs	563.89	(2568.91)	548.77	(2534.93)	15.12	(23.25)	558.73	(2611.32)	5.17	(19.07)	523.33	(2577.07)	40.57	(39.89)
Sum of costs	2507.88	(2864.38)	2467.00	(2807.64)	40.89	(28.29)	2491.31	(2911.74)	16.58	(21.78)	2393.36	(2836.42)	**114.5	(47.70)

*p-value of paired *t*-test/bootstrapping of differences between complete and incomplete cost collection <0.10.

**p-value of paired *t*-test/bootstrapping of differences between complete and incomplete cost collection <0.05.

^a^Alternative 0, complete cost collection.

^b^Alternative 4, incomplete cost collection, omitted quarter 2, replaced by quarters 1 and 3.

^c^Alternative 5, incomplete cost collection, omitted quarter 3, replaced by quarters 2 and 4.

^d^Alternative 6, incomplete cost collection, omitted quarters 2 and 3, replaced by quarters 1 and 4.

In the case of incomplete data collection and interpolation, there were three significant differences in comparison with complete data collection. Drug costs differed when applying Alt 5 and Alt 6; costs of ambulatory clinics in hospital differed when applying Alt 6; the sum of direct healthcare costs differed when applying Alt 4. In addition, most of the differences between complete and incomplete cost collection were larger than the differences from extrapolation.

Total cost estimation from incomplete data collection in the case of two data collections was significantly lower independent of whether extrapolation (3.9%) or interpolation (4.6%) was used. Interpolation and extrapolation in the case of omitting information from the second or third quarter showed no difference.

Table 6 illustrates the precision regarding change in standard deviation due to incomplete data collection. In some cases, we found smaller and, in some cases, larger standard deviations than in complete data collection. For the sum of direct healthcare costs, the largest increase was 6.45%, for the sum of direct non-healthcare costs 1.65%, and for the sum altogether 1.65%.

**Table 6:** Percentage change in variance of the estimate: complete cost collection vs. incomplete cost collection

	**Complete cost collection**	**Incomplete cost collection**											
		**Extrapolation**						**Interpolation**					
	**Alt 0**^**a**^	**Alt 1**^**b**^		**Alt 2**^**c**^		**Alt 3**^**d**^		**Alt 4**^**e**^		**Alt 5**^**f**^		**Alt 6**^**d**^	
**Cost category**	**(SD)**	**(SD)**	**shift**	**(SD)**	**shift**	**(SD)**	**shift**	**(SD)**	**shift**	**(SD)**	**shift**	**(SD)**	**shift**
Sum of direct healthcare costs	(936.67)	(946.22)	1.02	(962.29)	2.74	(997.06)	6.45	(917.58)	−2.04	(962.72)	2.78	(936.79)	0.01
Sum of direct non-healthcare costs	(2568.91)	(2534.07)	−1.36	(2589.39)	0.80	(2598.16)	1.14	(2534.93)	−1.32	(2611.32)	1.65	(2577.07)	0.32
Sum of costs	(2864.38)	(2827.43)	−1.29	(2882.21)	0.62	(2893.05)	1.00	(2807.64)	−1.98	(2911.74)	1.65	(2836.42)	−0.89

^a^Alternative 0, complete cost collection.

^b^Alternative 1, incomplete cost collection, omitted quarter 2, replaced by quarter 3.

^c^Alternative 2, incomplete cost collection, omitted quarter 3, replaced by quarter 4.

^d^Alternative 3, incomplete cost collection, omitted quarters 2 and 3, replaced by quarter 4.

^e^Alternative 4, incomplete cost collection, omitted quarter 2, replaced by quarters 1 and 3.

^f^Alternative 5, incomplete cost collection, omitted quarter 3, replaced by quarters 2 and 4.

^g^Alternative 6, incomplete cost collection, omitted quarters 2 and 3, replaced by quarters 1 and 4.

### The consequence on sample size calculation

Incomplete data collection will universally lead to increasing variance of the estimate [13,14]. As the variance and standard deviation influence sample size calculation, we modelled the impact of a 1% increase in standard deviation on sample size, called elasticity (ε). Mathematical calculations are shown in detail in Appendix 1. In the case of a single outcome (mean difference in costs), the sample size has to increase by 2% if the standard deviation increases by 1%. In the case of cost-effectiveness research, the increase in sample size is smaller. If the standard deviation increases by 1%, the sample size has to increase by $2\frac{b}{d+b}\%$. As b >0 and d >0, the elasticity for cost-effectiveness is lower than for a single outcome.

For the sum of costs, the largest increase was 1.65% in the case of Alt 5. This would lead to an increase in sample size of 3.3%, i.e. about 12 patients (from planned 340 to 352).

The necessary time to conduct the quarterly interview including set-up time was estimated at 0.5 h. The time for initial and final assessment was estimated at 3 h per assessment. Incomplete data collection would save between 170 h (one omitted quarter) and 340 h (two omitted quarters) compared with 84 h, which are still required for two quarterly interviews and initial and final assessment for additional patients.

## Discussion

The aims of this paper were to define suitable resource categories for incomplete cost data collection, to assess the validity of alternative strategies, and to point out the consequences with respect to efficiency.

To determine the resource category suitable for incomplete data collection, inherent recall bias and mean differences between quarters should be assessed. In the case of minor recall bias, Clarke et al. recommend only the collection of complete data by extending recall windows to ensure that no information is lost and data are still collected less frequently [13]. Applied to our study, this means that data collection in the first quarter for the previous time period of 3 months and in the fourth quarter for the previous 9 months would be preferable to incomplete data collection. Recall may be influenced by the frequency and severity of events, [9,11] so that less frequent visits and more serious events improve the memory of resource use.

It became apparent that it is not appropriate to collect data on hospitalisation, rehabilitation and care insurance benefits using an incomplete algorithm, but to account for the entire target period by using longer recall periods. These cost categories were both relevantly different between quarters and the associated events were less frequent or more serious. Inpatient care indicates a serious event, and patients who had at least one admission to hospital had on average 1.9 stays per year (Table 2). As rehabilitation only occurs once a year, it is a rare event; only five patients reported two stays per year. Care insurance benefits are granted by care insurance funds, following a lengthy needs assessment process. For these cases, recall bias is hardly likely. Bhandari et al. also stated in their review that hospitalisation is a salient event that may be gathered accurately by applying longer recall periods [9] without an appreciable increase in recall bias. In addition, patient reports on hospital stays could be cross-validated against electronic hospital records where these exist. Clarke et al. recommended trading off recall bias against information loss, which would be caused by incomplete data collection. Only if the degree of variation introduced by incomplete data collection is smaller than the bias introduced by recall error should a short time period for cost assessment be preferred [13].

Attention should be paid to the fact that care insurance benefit is a proxy for informal care. If costs incurred for informal care are determined by the hours of care provided by relatives, neighbours or friends, incomplete data collection can be used in a similar way to the collection of outpatient nursing services or paid household help.

The resource categories physiotherapy, ambulatory clinic in hospital, medication, consultations (physicians), outpatient nursing service and paid household help are deemed to be appropriate for incomplete data collection for several reasons. First, there is no evidence for differences in mean costs between the middle quarters (quarters two and three) (Table 3). Second, the portion of these costs is about 30% of total costs, so that an inaccuracy of 5% causes a difference of only 1.5%. The main cost-driving events are hospitalisation and rehabilitation accounting for about 70%. Ridyard et al. [8] advise in their systematic review the balancing of resource use data collection between the main cost driving events, the frequency of data collection and the burden on the researcher. Third, existing differences in mean costs between quarter one and all the other quarters do not become important in the case of extrapolation. Although cost data from quarter one are employed in the case of interpolation, only the sum of direct healthcare costs differed significantly from complete cost collection (Table 5, Alt 4) when data were collected three times.

The lower costs in the resource categories physiotherapy, ambulatory clinic in hospital, medication, consultations (physicians), outpatient nursing service, and paid household help in quarter one (Table 3) can be explained by increased use of rehabilitation and hospital admissions during this time, which supplants ambulatory resource use.

Significant differences in mean costs between quarter four and quarters two or three occur in the case of medication data (Table 3). This reflects the drug regimen guidelines [35,36] for the acute coronary syndrome, which recommend the use of clopidogrel, an antiplatelet drug, as follow-up treatment for up to 9 months only.

In our analysis, extrapolation turned out to be the better instrument for replacing the omitted periods, as quarter one showed consistently lower costs. Data from quarter one were not used for extrapolation, but for interpolation. Therefore, mean differences between complete and incomplete cost collection from extrapolation were mostly smaller than differences from interpolation (Tables 4 and 5). Furthermore, medication data proved to be only suitable for omitting quarter two and replacing by quarter three, as quarter four is not a representative quarter.

A comparison of our results is restricted by the lack of publications of empirical analyses regarding incomplete data collection. In a study of 174 patients with a stable chronic disease (fibromyalgia and low back pain), Goossens et al. showed no significant differences between multiple time frames of incomplete data collection [14]. Thus, the authors concluded that, for patients with chronic diseases, incomplete cost data collection poses no problem for economic evaluations. They compared the differences in median by Wilcoxon’s signed rank test but not in arithmetic means, and they did not distinguish between different cost categories. As the comparison of means is central to any economic evaluation, non-parametric tests that address differences in the median and analyses of log-transformed costs that address differences in the geometric means are not well suited for this purpose [37]. However, in comparison with our study, the sample size of 174 participants was even smaller, which poses severe limitations with regard to the statistical power of the analysis.

Nevertheless, the authors indicated that, in the case of acute diseases, randomised clinical trials and chronic diseases with seasonal effects, the necessary assumptions of agreement between the different time periods could not be met. Clarke et al. [13] argued that irregular consumption patterns add estimation error. Seasonal effects only become important if the time frame of recruitment is relatively short. If recruitment or the start of the intervention cover a 1-year period, seasonal effects occur for individuals but not for groups. As the comparison of arithmetic means between groups is central to any economic evaluation, group estimates have to be valid, but the results for individuals may differ from each other [15]. Our analysis shows that, in the case of acute myocardial infarction with a 1-year follow-up, several kinds of resource categories are more appropriate for incomplete cost data collection than others. Generalisation of our findings is limited to the elderly population with acute diseases, followed by a chronic course associated with a continuous treatment scheme, as patients with acute myocardial infarction have similar patterns in the long-term course of disease and treatment. Applying incomplete data collection, several points have to be considered when choosing the method (inter- or extrapolation) and omitting quarters. Only those periods can be omitted for which it can be assumed that they are representative of other periods. Equally, only periods for which one may assume that they represented omitted periods can be used to replace the omitted periods. For other studies, the choice of omitted periods may depend on the disease and the intervention so that these assumptions must be tested in pilot studies or based on expert opinions or literature research.

It is important to reduce the burden on study participants, especially in older participants, by decreasing the frequency of data collection. Because this can be achieved by incomplete data collection or extending recall windows, one should carefully consider and differentiate which time frame and method of cost data collection are appropriate for the respective resource category. As an example, Heinrich et al. [16] assessed resource use by employing different time frames for the respective resource category, whereas Hakkaart-van Roijen et al. [17] and Kimman et al. [18] did not distinguish between different resource categories.

A further problem resulting from incomplete cost collection arises from the withdrawal of informed consent or death of the study participants, as it can be assumed that missing data increase because of longer time periods between data collection. For this reason, we recommend not omitting the first quarter.

Although incomplete cost collection will universally lead to increasing variance of the estimate, [13,14] we only found partially larger standard deviations than in complete data collection. Goossens et al. exclusively found smaller standard deviations, which they attributed to random error, and they recommended including ‘more’ patients [14]. To our knowledge, no calculation concerning increasing standard deviations and sample size has been published so far. When we assume that a suitable resource category will be collected incompletely, our estimate requires a larger sample size of about 3% at most. Nevertheless, more time would be saved as a result of incomplete data collection than extra time required for assessing additional patients. Furthermore, the burden on study participants and clinical investigators can be diminished through the economic data collection effort. When conducting economic analysis alongside clinical trials by means of incomplete data collection, sample size calculation has to be modified.

## Conclusions

In economic evaluation, cost data can be collected efficiently by reducing the frequency of data collection. This can be achieved by data collection for shorter periods, by implication incomplete data collection, or extending recall windows so that data are collected completely. To minimise bias by recall error or information loss, one has to consider carefully which resource category is suitable for incomplete data collection. In our analysis, cost estimates per year for ambulatory healthcare and non-healthcare services in terms of three data collections was as valid and accurate as a four complete data collections. Applying incomplete data collection, it should be considered that only periods are suitable to be omitted that have similarity to other periods. The choice of the periods depends on the disease, treatment guidelines and the intervention.

When using the method of incomplete data collection sample, size calculation has to be modified because of increased variation. This approach is suitable to lower the burden and costs for the study participants and investigators in economic evaluation alongside clinical trials. Further empirical analysis regarding the validity of incomplete cost collection must be performed in order to improve the already existing practice of incomplete cost data collection.

## Appendix 1

The equation for sample size calculation for a single outcome, thus as costs (or effects) between groups is [33]

$$n>\frac{{\left(z_{\alpha /2}+z_{\beta }\right)}^{2}\left(\sigma_{\mathrm{CT}}^{2}+\sigma_{\mathrm{CC}}^{2}\right)}{\Delta {\tilde{E}}^{2}}$$

where α type I error, the probability of falsely rejecting the null hypothesis when in fact a true difference exists$z_{\alpha /2}$ standardised normal deviate, such for α=0.05, $z_{\alpha /2}$(two-sided)=1.96β type II error, the probability of not rejecting the null hypothesis when it is false$z_{\beta }$standardised normal deviate, such for β=0.10, $z_{\beta }$(one-sided)=1.28$\sigma_{\mathrm{CT}}^{2}$variance of the costs in the intervention (trial) group$\sigma_{\mathrm{CC}}^{2}$ variance of the costs in the control group$\Delta \tilde{Ε}$ difference in costs between two groupsTo simplify the equation:${\left(z_{\alpha /2}+z_{\beta }\right)}^{2}=a^{2}$$\left(\sigma_{\mathrm{CT}}^{2}+\sigma_{\mathrm{CC}}^{2}\right)=b^{2}$$\Delta {\tilde{Ε}}^{2}=c^{2}$

$$n>\frac{a^{2}b^{2}}{c^{2}}$$

The equation for ε is

$$\varepsilon =\frac{\partial f}{\partial b}\cdot \frac{b}{f}$$

=2ba2c2⋅ba2b2c2

=2If the standard deviation increases by 1 percent, the sample size has to increase by 2 percent.The equation for sample size calculation for cost-effectiveness is [33]

$$n>{\left[\frac{\left(z_{\alpha /2}+z_{\beta }\right)\left(R_{c}\sqrt{\sigma_{\mathrm{ET}}^{2}}+\sqrt{\sigma_{\mathrm{CT}}^{2}+\sigma_{\mathrm{CC}}^{2}}\right)}{\Delta \tilde{C}-R_{c}\Delta \tilde{E}}\right]}^{2}$$

where$\sigma_{\mathrm{ET}}^{2}$variance of the effects in the intervention (trial) group$\sigma_{\mathrm{EC}}^{2}$ variance of the effects in the control group$\sigma_{\mathrm{CT}}^{2}$variance of the costs in the intervention (trial) group$\sigma_{\mathrm{CC}}^{2}$ variance of the costs in the control group$\Delta \tilde{C}$ difference in costs between two groups$\Delta \tilde{E}$ difference in effects between two groups$R_{c}$„ ceiling“value (maximum acceptable value) of ICER (incremental cost-effectiveness ratio)To simplify the equation:$\left(z_{\alpha /2}+z_{\beta }\right)=a$$\sqrt{\sigma_{\mathrm{CT}}^{2}+\sigma_{\mathrm{CC}}^{2}}=b$$R_{c}\sqrt{\sigma_{\mathrm{ET}}^{2}+\sigma_{\mathrm{EC}}^{2}}=d$$\Delta \tilde{C}-R_{c}\Delta \tilde{E}=c$

$$n>{\left[\frac{a\left(d+b\right)}{c}\right]}^{2}$$

The equation for ε is

$$\varepsilon =\frac{\partial f}{\partial b}\cdot \frac{b}{f}$$

$$=\frac{2a\left(d+b\right)}{c}\cdot \frac{a}{c}\cdot \frac{b}{{\left[\frac{a\cdot \left(d+b\right)}{c}\right]}^{2}}$$

$$=2\frac{b}{d+b}$$

If the standard deviation increases by 1 percent, the sample size has to increase by$=2\frac{b}{d+b}$ percent. Since b>0 and d>0, the elasticity for cost-effectiveness is lower than for a single outcome.

## Competing interests

The authors declare that they have no competing interests.

## Authors’ contributions

HS determined unit prices and calculated costs, performed the statistical analyses, calculated the elasticity and drafted the manuscript. CM conceived the KORINNA study and participated in its design and coordination. RW collected the data and coordinated the KORINNA study. RH conceived the KORINNA study, participated in its design and coordination and helped to draft the manuscript. All authors read and approved the final manuscript.

## Pre-publication history

The pre-publication history for this paper can be accessed here:

http://www.biomedcentral.com/1472-6963/12/318/prepub
